# Development of target-biased scoring functions for protein-ligand docking

**DOI:** 10.1186/1758-2946-4-S1-P35

**Published:** 2012-05-01

**Authors:** Michael Scharfe, Martin Pippel, Wolfgang Sippl

**Affiliations:** 1 Institute of Pharmacy, Pharmaceutical Chemistry, University Halle-Wittenberg, Halle, 06120, Germany

## 

Accurate scoring of protein-ligand interactions for docking, binding-affinity prediction and virtual screening campaigns is still challenging. Despite great efforts, the performance of existing scoring functions strongly depends on the target structure under investigation. Recent developments in the direction of target-class-specific scoring methods and machine-learning-based procedures reveal significant performance improvement in binding mode and affinity prediction.

However, there is no open-source framework that combines such sophisticated techniques with molecular docking algorithms to make them simply applicable for virtual screening. Therefore, the aim of this work is the implementation of general tools within the open-source framework ParaDockS [1] to obtain target-class-specific scoring functions. These scoring functions are based on knowledge-based atom-pair potentials which can be used in an additive manner (PMF-Score) to score protein-ligand complexes. Because ParaDockS already includes algorithms for protein-ligand docking, every new obtained scoring function can be immediately applied.

Recently it was shown, that atom-pair potentials are also useful for training machine-learning models like support vector machines or random forests [2]. Such methods circumvent a particular functional form for the scoring function and thereby implicitly capture binding contributions that are hard to model explicitly. Our goal is to implement different machine-learning procedures within the ParaDockS framework to provide further possibilities for scoring protein-ligand complexes.

In a first test and validation study, we applied this workflow on kinase data sets, but in principle it is applicable to every target class with enough structural data. In further studies we want to obtain a set of different scoring functions that are biased to a certain target class and can be used for docking and scoring within ParaDockS.
